# Effects of Microclimate Condition Changes Due to Land Use and Land Cover Changes on the Survivorship of Malaria Vectors in China-Myanmar Border Region

**DOI:** 10.1371/journal.pone.0155301

**Published:** 2016-05-12

**Authors:** Daibin Zhong, Xiaoming Wang, Tielong Xu, Guofa Zhou, Ying Wang, Ming-Chieh Lee, Joshua A. Hartsel, Liwang Cui, Bin Zheng, Guiyun Yan

**Affiliations:** 1 Program in Public Health, College of Health Sciences, University of California at Irvine, Irvine, California, United States of America; 2 Key Laboratory of Prevention and Control for Emerging Infectious Diseases of Guangdong Higher Institutes, School of Public Health and Tropical Medicine, Southern Medical University, Guangzhou, People's Republic of China; 3 National Institute of Parasitic Diseases, Chinese Center for Disease Control and Prevention, Shanghai, People's Republic of China; 4 Department of Pathogen Biology, College of Basic Medical Sciences, Third Military Medical University, Chongqing, People's Republic of China; 5 Department of Entomology, the Pennsylvania State University, University Park, Pennsylvania, 16802, United States of America; Swedish University of Agricultural Sciences, SWEDEN

## Abstract

In the past decade, developing countries have been experiencing rapid land use and land cover changes, including deforestation and cultivation of previously forested land. However, little is known about the impact of deforestation and land-use changes on the life history of malaria vectors and their effects on malaria transmission. This study examined the effects of deforestation and crop cultivation on the adult survivorship of major malaria mosquitoes, *Anopheles sinensis* and *An*. *minimus* in the China-Myanmar border region. We examined three conditions: indoor, forested, and banana plantation. Mean survival time of *An*. *sinensis* in banana plantation environment was significantly longer than those in forested environment, and mosquitoes exhibited the longest longevity in the indoor environment. This pattern held for both males and females, and also for *An*. *minimus*. To further test the effect of temperature on mosquito survival, we used two study sites with different elevation and ambient temperatures. Significantly higher survivorship of both species was found in sites with lower elevation and higher ambient temperature. Increased vector survival in the deforested area could have an important impact on malaria transmission in Southeast Asia. Understanding how deforestation impacts vector survivorship can help combat malaria transmission.

## Introduction

Malaria is a significant public health problem affecting predominantly vulnerable pregnant women and children in Africa [1–3]. Interestingly, in Southeast Asia, there has been a change in malaria epidemiology where the adult male population has borne a greater burden of disease [4, 5]. Although there is a strong correlation between malaria and poverty [6–9], malaria is both a cause and a consequence of poverty. Malaria’s devastating effects have historically been observed in countries of the Greater Mekong Subregion (GMS). Since the inception of the World Health Organization’s Mekong Malaria Program a decade ago, the malaria situation in the GMS has been greatly improved, reflected by the continuous decline in annual malaria incidence and deaths [1, 10]. However, as all countries within the GMS are moving towards malaria elimination, significant challenges remain, particularly in Myanmar, where the regional malaria burden is the heaviest [10]. Malaria epidemiology in this region has several characteristics, including being an epicenter of antimalarial drug resistance [10–13], high malaria transmission in the forested fringe areas [4, 14] and in the remote border region [15–17], and cross-border malaria introduction due to human movement [11]. For example, over 90% of the imported *falciparum* malaria in China was introduced across the China-Myanmar border [18, 19]. As the GMS countries aim at malaria elimination by 2030 [20], it is crucial to examine the current situations of malaria epidemiology and vector biology in the high-risk border region so that optimal elimination strategies can be developed.

Vector control has historically been the most effective method to reduce malaria transmission. It remains the most important tool available, but its effectiveness relies on a thorough understanding of vector biology and their interactions with the environment. Due to human population expansion and an increasing demand for food supply, deforestation has become a very serious problem in the China-Myanmar border area [21–23]. Consequently, previously forested areas are often converted to lands for subsistence and cash crops. The changes in the environmental conditions in the area have been shown to alter malaria vector species composition [24–27] and in turn, malaria transmission. Different vector species vary in their feeding behaviors and vector competence [28–30]. However, the ecological mechanisms underlying malaria vector species succession are not well understood.

The objective of the present study is to determine the effects of land use and land cover on the adult survivorship of major malaria vector species in the China-Myanmar border area using the life-table experiments. We found that mosquito survivorship was strongly influenced by the microclimatic conditions such as ambient temperature, which is directly affected by land use and land cover. This information is useful for predicting the impact of environmental and climate changes on vectorial capacity.

## Materials and Methods

### Ethics Statement

No specific permits were required for the described field studies. For mosquito collection in banana fields, oral consent was obtained from field owners in each location. These locations were not protected land, and the field studies did not involve endangered or protected species. The use of mice in mosquito blood-feeding was performed in strict accordance with the recommendations in the Guide for the Care and Use of Laboratory Animals of the National Institutes of Health. All of the animals were handled according to approved institutional animal care and use committee (IACUC) protocols (#2008–2774) of University of California at Irvine.

### Study Sites

**Fig 1:**
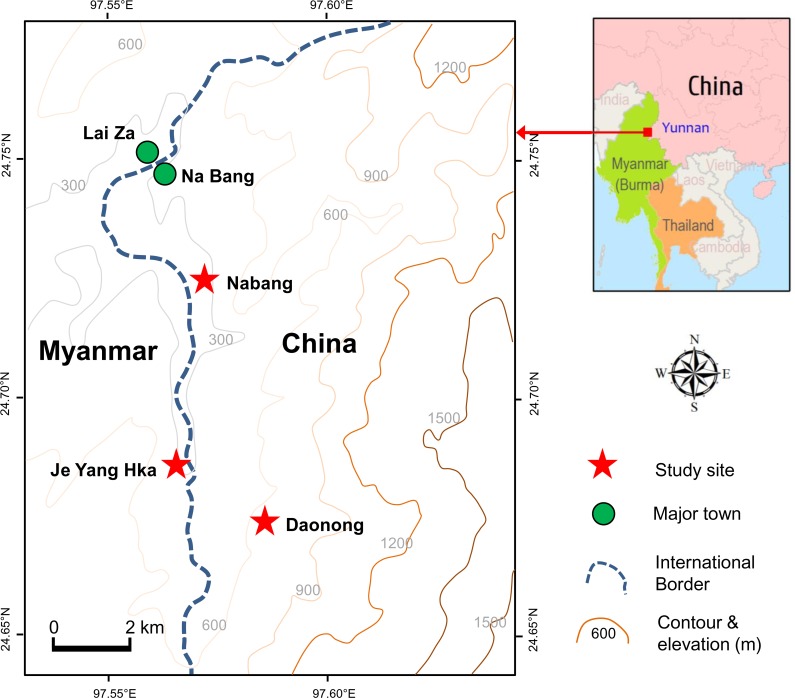
Study area and sampling sites in the China-Myanmar border region.

The study was conducted in the China-Myanmar border region (24°44´12"N, 97°36´9"E). The study sites were located in Kachin State, Myanmar and Yingjiang County of Yunnan Province, China (Fig 1). The study area covered an area of about 100 km^2^, including two villages (Nabang and Daonong) in China and one site near the town of Laiza, Kachin State in Myanmar. The two sites in China differ in elevation with Nabang at 240 m and Daonong at 660 m above sea level. The site in Myanmar, Je Yang Hka, is about 5 km from Nabang with an elevation of ~200 m above sea level. The study area is a hilly area, with mountains being covered mainly by forest or maize/banana plantations after deforestation and the valley areas being covered by banana plantations. Rubber, black pepper, and banana farming are the predominant agricultural activities, and food crops of mostly maize are cultivated at a very small scale. The average annual rainfall in the past 30 years was 1,464 mm and average monthly ambient temperature was 19.3°C. The mosquito larvae habitats in the study area included ponds, puddles, swamps, and other sources of stagnant aquatic habitats.

### Effects of Land Use and Land Cover on Mosquito Survival

We examined three land cover scenarios: banana planation, forest, and indoor environment within typical local houses. The study was conducted from May to July in 2014, during the peak malaria transmission season in this area [31, 32]. The peak vector abundance was occurred from April to July each year [33, 34]. A forested area is defined as an area with >60% tree canopy coverage measured by ground shade area, and the vegetation was mainly subtropical evergreen broad-leaf rainforest with some deciduous trees in the canopy layer. The banana plantation was an area of banana plants planted two years prior to this study, and the canopy coverage was about 40% measured by ground shade area. Typical local houses have mixed brick and wood/bamboo structures, with brick/concrete walls on the ground floor and wood/bamboo walls on the elevated floor. Windows are usually not screened. Residents usually spend the night upstairs but may take naps downstairs during noon time. The major vector mosquito species are *Anopheles minimus* (forested area) and *Anopheles sinensis* (deforested area).

Approximately 5,000 Anopheline mosquito larvae were collected from local habitats and reared to adults in an insectary located in Nabang. To avoid using mosquitoes from one single female, we collected no more than 50 third- to fourth-instar larvae per habitat and reared them to adults under the same conditions. Emerged adults were identified to species or species complex using published morphological keys of Dong [35]. Species-specific polymerase chain reactions (PCR) were used for confirmatory identification of the species for a subset of adult mosquitoes [36–39]. Newly emerged adults were used for life-table studies. Briefly, 50 female and 50 male adult mosquitoes within 24 h post-emergence were placed in a cylindrical cage of 20 cm in diameter and 30 cm in height. The cage was covered with nylon mesh to prevent the escaping of mosquitoes. Four replicates were used for each of the three environments. In the forested environment and banana plantation, the cages were suspended under a tree or banana leaf, 2 m above the ground. In the indoor environment, mosquito cages were hung in the middle of the living room, also 2 m above the ground. A plastic cap filled with water was hung directly above the cages to prevent ants from entering the cages. Plastic covers were placed on the top of the cages to protect cages from rains. Mosquitoes were provided with 10% sucrose and one mouse in each cage was used to blood feed mosquitoes for approximately 30 minutes every morning. The cages were examined daily for the numbers of surviving and dead mosquitoes, and dead mosquitoes were then removed. HOBO data loggers (Onset Computer Corp., Bourne, MA) were placed inside the cages to record hourly temperature, relative humidity, and light intensity every min during the entire duration of the experiment. The HOBO data logger is a compact, battery-powered device equipped with an internal microprocessor, data storage, and one or more sensors, which can be used to track environmental temperature, relative humidity and light intensity. Life-table studies were conducted for *An*. *sinensis* and *An*. *minimus*, the two predominant malaria vector species in the study area.

### *Anopheles* Mosquito Life-Table Experiments in High- and Low-Elevation Area

To further confirm the effects of microclimate conditions on mosquito survivorship, we conducted life-table studies in an indoor environment at two sites differing in elevation and in indoor air temperature. The study was conducted in Nabang (elevation 240 m) and Daonong (elevation 660 m) villages, from April to June in 2012. The same procedures described above were used for larval sample collection, mosquito rearing, cage placement conditions, and blood feeding to determine daily survivorship. Four replicates were used in each village. HOBO data loggers were placed inside the same houses to measure hourly indoor temperature, relative humidity, and light intensity at the two study sites for the entire duration of the experiment.

### Statistical Analysis

Data were analyzed to address the following questions: 1) Do land use and land cover significantly affect mosquito survivorship? We addressed this question using Kaplan-Meier survival analysis [40] to determine the variation in daily survivorship among mosquitoes placed in different land use and land cover types, or between two sites of different elevations. 2) Do land use and land cover significantly affect the microclimatic conditions of local niches where adult mosquitoes were tested for survivorship? Daily average, minimum, and maximum temperatures and relative humidity were calculated from the hourly record. Analysis of variance (ANOVA) with repeated measures was used to determine the differences in these microclimatic variables across different land use and land cover types. The post hoc, Tukey’s honestly significant difference (HSD) test was used to determine which groups significantly differed from each other. Tukey’s HSD procedure was developed specifically to account for multiple comparisons and maintains an experiment-wise error rate at the specified level [41]. 3) Do mosquito species (*An*. *sinensis* and *An*. *minimus*) differ in their response to the microclimatic conditions in survivorship? Kaplan-Meier survival analysis was used to compare the two mosquito species under the same environmental conditions. A log-rank test was used to determine the significance of difference between two survival curves. All analyses were conducted using JMP statistical software [42].

## Results

### Community Structure of *Anopheles* Mosquitoes

Among the 7,736 adult *Anopheles* mosquitoes emerging from the larval collections, a total of 18 species were identified (Table 1). The most abundant species (complex) was *An*. *sinensis* (50.5%), followed by *An*. *minimus* (21.5%), *An*. *barbirostris* (10.8%) and *An*. *maculatus* (6.3%). Morphological identification of the mosquitoes was confirmed by species-specific PCR. The subsequent life-table studies were conducted with the two most abundant malaria vector species, *An*. *sinensis* and *An*. *minimus*.

**Table 1:** Composition of Anopheles adult mosquito rearing from larvae collected in China-Myanmar border region.

*Species*	*Female*	*Male*	*Total*	*Percentage (%)*
*Anopheles sinensis *	2,091	1,815	3,906	50.49
*Anopheles minimus*	894	770	1,664	21.51
*Anopheles barbirostris *	477	360	837	10.82
*Anopheles maculatus*	300	186	486	6.28
*Anopheles splendidus*	168	69	237	3.06
*Anopheles peditaeniatus*	87	96	183	2.37
*Anopheles vagus*	42	56	98	1.27
*Anopheles crawfordi*	42	36	78	1.01
*Anopheles kochi*	42	33	75	0.97
*Anopheles culicifacies *	25	21	46	0.59
*Anopheles jetporiensis*	20	15	35	0.45
*Anopheles barbumbrosus*	21	10	31	0.40
*Anopheles tessellatus*	14	6	20	0.26
*Anopheles hyrcanus*	10	8	18	0.23
*Anopheles annularis*	6	6	12	0.16
*Anopheles annularis*	3	1	4	0.05
*Anopheles messeae*	2	1	3	0.04
*Anopheles lesteri *	1	2	3	0.04
Total	4,245	3,491	7,736	100

### Effects of Land Use and Land Cover on Mosquito Survivorship

**Fig 2:**
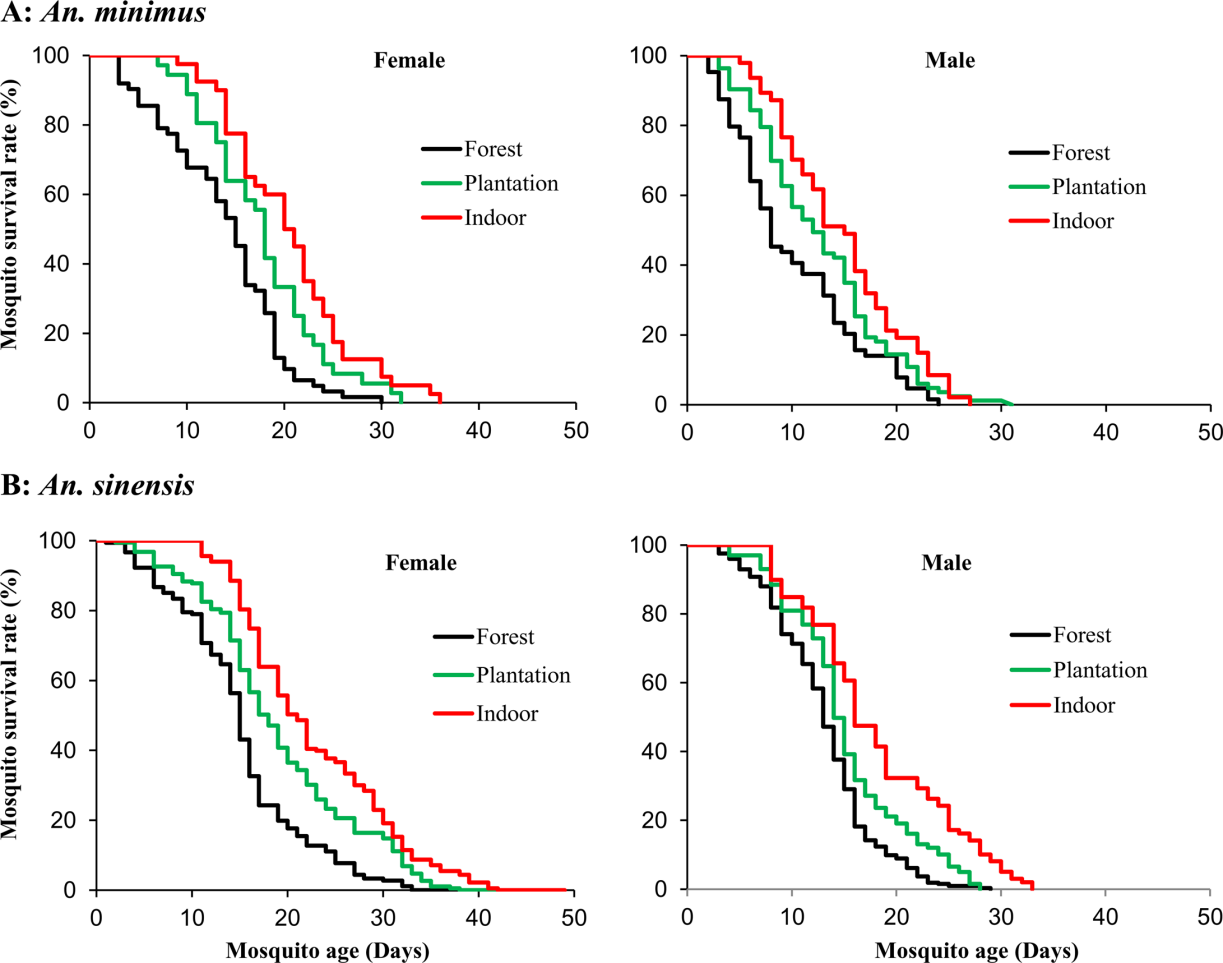
Kaplan-Meier survival analysis. Impact of land use and land cover on survivorship of *Anopheles minimus* (top panel) and *An*. *sinensis* (bottom panel) adult mosquitoes. Three land use and land cover types were tested: indoor, banana planation and forest.

Survivorship of adults reared from wild-caught larvae and pupae was examined in three different environments: indoor, plantation, and forest (S1 Table). Female mosquitoes placed indoors survived significantly longer than those in banana plantation and forest for both *An*. *minimus* (F_2,9_ = 13.5, P < 0.0014) and *An*. *sinensis* (F_2,9_ = 33.6, P < 0.0001) (Fig 2). The mean survival duration of female *An*. *minimus* mosquitoes were 21.6, 18.8 and 14.8 days in indoor, banana plantation and forest, respectively (Table 2). A similar result was found in female *An*. *sinensis* mosquitoes in different land use and land cover settings. Male mosquitoes lived for a significantly shorter period of time than females for both *An*. *minimus* and *An*. *sinensis*, but the pattern of survivorship in indoor, banana plantation, and forest environment was the same as the females (Fig 2). The daily survival rate ranged from 0.88 to 0.91 for females and 0.84 to 0.89 for males (Table 2).

**Table 2:** Comparisons of mean and median survivorship of *Anopheles minimus* and *An*. *sinensis* adults in different land use and land cover conditions. Values are shown as mean ± standard deviation.

Species	*An*. *minimus*			*An*. *sinensis*		
Study site	Forest	Plantation	Indoor	Forest	Plantation	Indoor
**Female survival time (days)**						
Mean±Standard deviation	14.8±1.1A*	18.8±1.5 A	21.6±2.6 B	15.0±1.3A	19.0±1.3 B	22.7±1.4 C
Median (95% confidence interval)	16 (14–17)	19 (15–20)	21 (18–23)	15 (14–16)	18 (16–19)	21 (19–22)
**Male survival time (days)**						
Mean±Standard deviation	11.2±1.3 A	13.8±1.9 AB	15.9±1.5 B	14.1±1.6 A	16.2±1.7 AB	19.1±2.4 B
Median (95% confidence interval)	9 (8–12)	13 (11–16)	16 (13–18)	14 (14–15)	15 (15–16)	17 (17–20)
**Female daily survival rate (%)**						
Mean±Standard deviation	88.2±14.6 A	90.2±13.0 a	91.1±13.2 A	87.9±13.9 A	88.1±14.9 A	91.4±10.5 A
**Male daily survival rate (%)**						
Mean±Standard deviation	85.6±16.2 A	87.1±13.3 a	88.7±16.3 A	83.6±16.9 A	87.4±14.3 A	89.4±12.0 A

* Values marked by different letters (A, B, and C) within the same row, for the same species, are significantly different at a level of 0.05 (Tukey-Kramer HSD test).

### Effects of Land Use and Land Cover on Microclimatic Conditions

**Fig 3:**
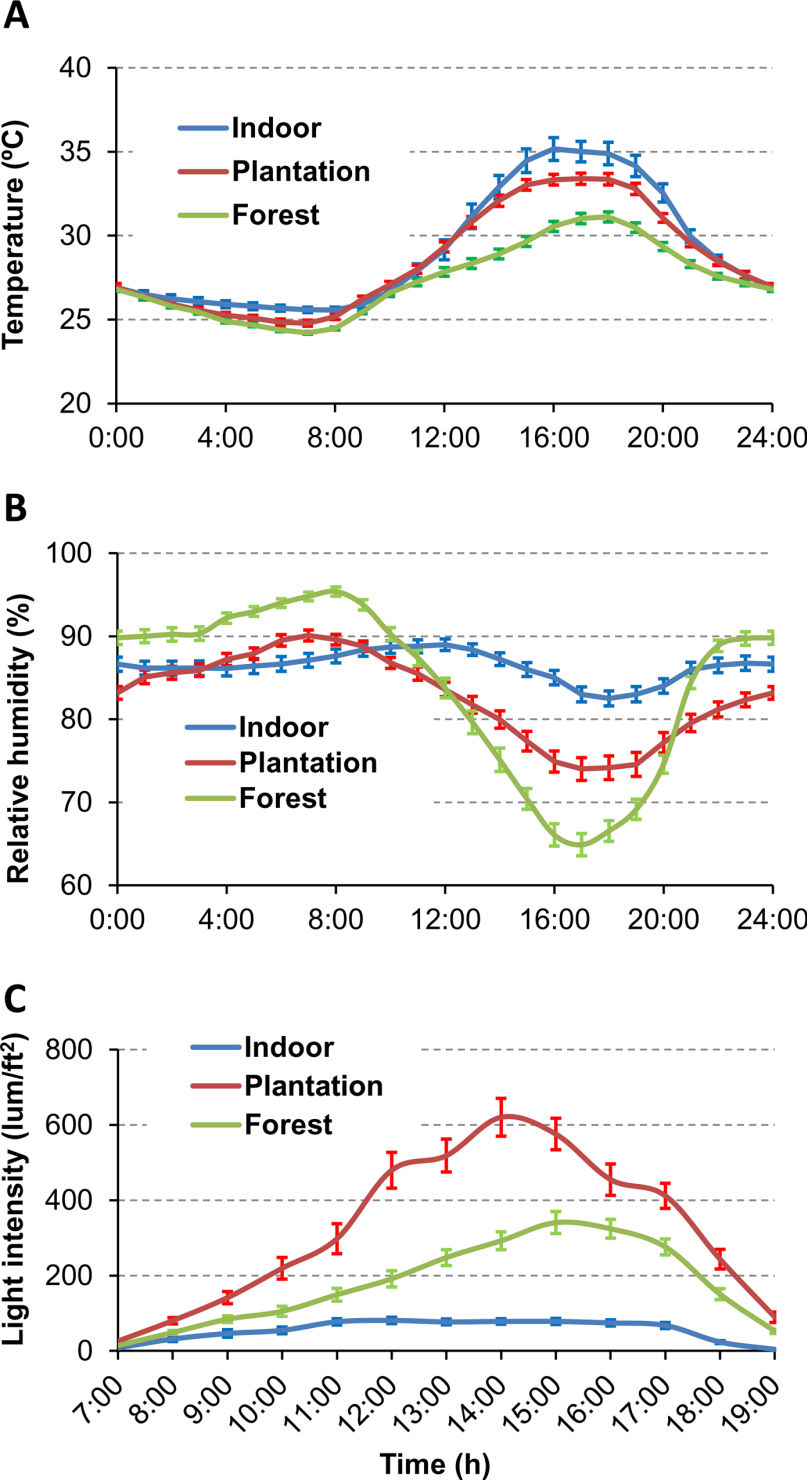
Daily dynamics of microclimatic conditions. Dynamics of hourly indoor temperature (**A**), relative humidity (**B**) and light intensity (C) under three land use and land cover conditions.

During the experiment period, the average ambient temperature in the forest was significantly lower than in the indoor environment (27.1 vs. 29.2°C, P < 0.0241) (Table 3), and the forest exhibited larger temperature fluctuations than those found in the indoor environment, as evidenced by larger differences between maximum and minimum temperature (Fig 3). The indoor environment exhibited the lowest light intensity. The relative humidity was high (>80%) and did not differ among these three land use and land cover conditions.

**Table 3:** Microclimate condition of mosquito niches tested in different land use and land cover conditions from May to July in 2014, in China-Myanmar border region. Values are shown as mean ± standard deviation.

Environment	Forest	Plantation	Indoor	F	P
Mean temperature (°C)	27.1±2.1A*	28.1±3.0 AB	29.2±2.6 B	3.94	0.0241
Mean relative humidity (%)	83.9±8.7 A	82.7±5.3 A	86.3±2.8 A	1.78	0.1763
Mean light intensity (lum/ft^2^)	175.0±112.0 A	319.8±202.6 B	53.8±28.4 C	12.7	<0.0001
Mean maximum temperature (°C)	31.8±4.0 A	30.1±2.2 AB	29.9±1.2 C	3.71	0.0294
Mean minimum temperature (°C)	23.8±1.5 A	24.3±0.4 B	26.9±0.6 B	70.0	<0.0001
Mean maximum relative humidity (%)	99.7±0.8 A	93.7±2.0 B	92.6±0.8 B	197.3	<0.0001
Mean minimum relative humidity (%)	54.9±13.5 A	71.1±6.2 B	77.4±1.5 C	43.2	<0.0001
Mean maximum light intensity (lum/ft^2^)	238.8±144.7 A	524.1±61.5 B	66.1±29.5 C	14.1	0.0017
Mean minimum light intensity (lum/ft^2^)	18.7±5.3 A	22.5±2.9 A	4.2±2.5 B	25.4	0.0002

* Values marked by different letters (A, B, and C) within the same row are significantly different at a level of 0.05 (Tukey-Kramer HSD test).

### Effects of Niche Temperature on Mosquito Survivorship

**Fig 4:**
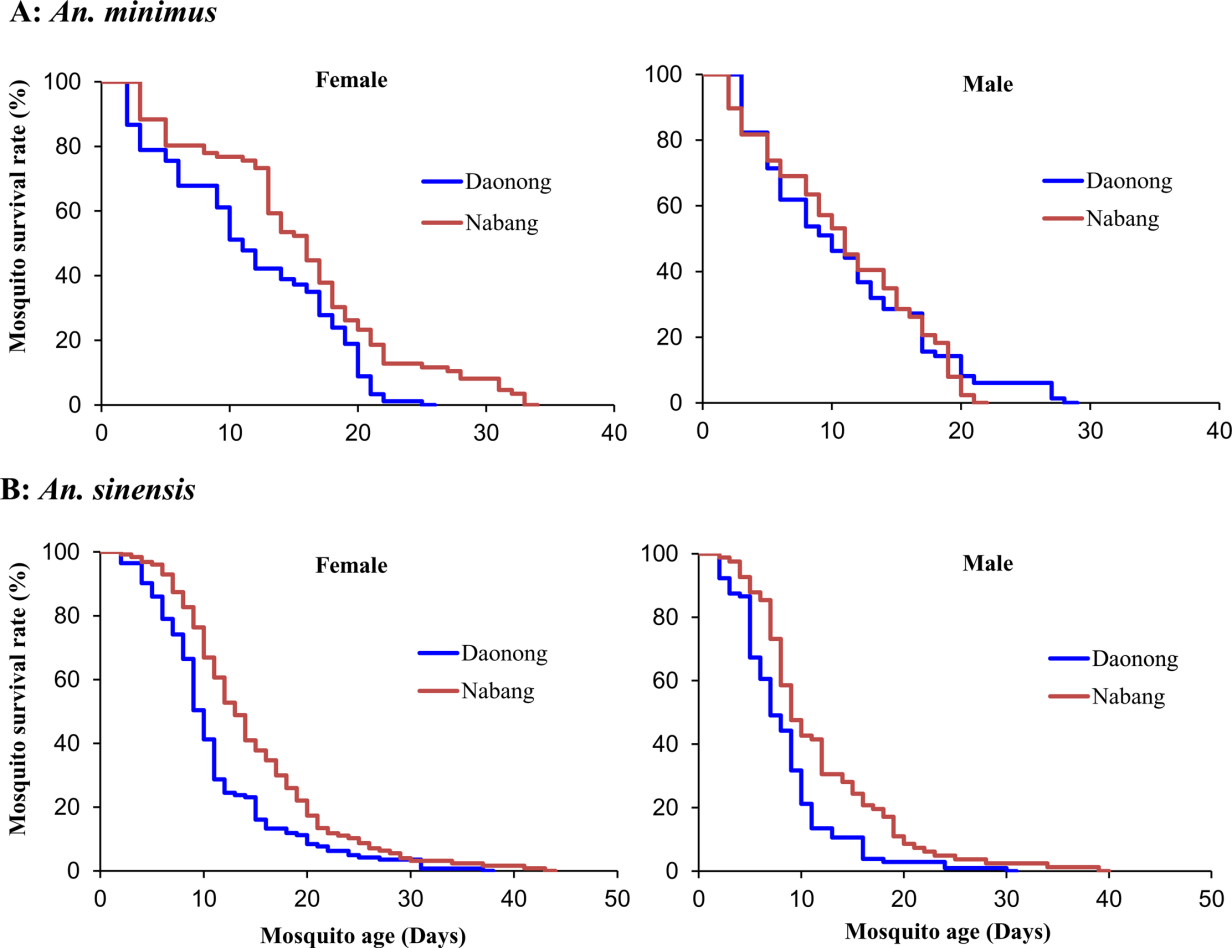
Life table survival analysis. Survivorship of *Anopheles minimus* (A) and *An*. *sinensis* (B) in two study sites differing in ambient temperature along the China-Myanmar border region.

To determine the impact of temperature on mosquito survival while maintaining similar light intensity, we examined mosquito survivorship in the indoor environment in two sites differing in elevation and air temperature only (S2 Table). The HOBO data loggers confirmed that the mean indoor temperature at the low-elevation site (Nabang) was 3.1°C higher than that at the high-elevation site (Daonong) (29.3 vs. 26.2, F_1,46_ = 29.5, P <0.0001; Table 4). There was no significant difference in the average indoor relative humidity between the two sites (Table 4). We found that female mosquitoes placed in houses at the low-elevation site (Nabang village) survived significantly longer than those at the high-elevation site (Daonong village) for both *An*. *minimus* (χ^2^ = 7.32, df = 1, P <0.01) and *An*. *sinensis* (χ^2^ = 13.9, df = 1, P <0.001) (Fig 4). For *An*. *minimus*, the mean longevity was 15.5 days in Nabang and 11.7 days in Daonong. For *An*. *sinensis*, the mean longevity was 14.7 days in Nabang and 11.1 days (female) in Daonong (Table 5). The same trend held for males although they died significantly faster than females (Table 5).

**Table 4:** Indoor climate condition in sites with different elevations in China-Myanmar border region. Values are shown as mean ± standard deviation.

Study site	Daonong (660 m)	Nabang (240 m)	F	P
Mean temperature (°C)	26.2±1.5 A*	29.3±2.3 B	29.5	<0.0001
Mean relative humidity (%)	78.6±3.9 A	77.6±7.6 B	0.36	0.5491
Mean maximum temperature (°C)	31.1±3.2 A	34.7±2.7 B	16.2	0.0002
Mean minimum temperature (°C)	23.2±0.4 A	25.8±0.8 B	197.9	<0.0001
Mean maximum relative humidity (%)	93.2±1.8 A	92.4±2.3 A	1.7	0.1899
Mean minimum relative humidity (%)	48.3±7.9 A	51.6±11.8 A	1.3	0.2631

* Values marked by different letters within the same row are significantly different at a level of 0.05 (Tukey-Kramer HSD test).

**Table 5:** Mean and median survivorship of *Anopheles minimus* and *An*. *sinensis* adults in indoor conditions in two sites with different elevations. Values are shown as mean ± standard deviation.

Species	*An*. *minimus*		*An*. *sinensis*	
Study site	Daonong (660 m)	Nabang (240 m)	Daonong (660 m)	Nabang (240 m)
**Female survival time (days)**				
Mean ± Standard deviation	11.7 ± 1.7 A*	15.5 ± 0.7 B	11.1 ± 1.1 A	14.7 ± 0.7 B
Median (95% confidence interval)	11 (10–12)	16 (14–17)	10 (9–11)	13 (12–14)
**Male survival time (days)**				
Mean ± Standard deviation	11.0 ± 0.8 A	11.1 ± 0.4 B	8.7 ± 1.0 A	11.7 ± 1.3 B
Median (95% confidence interval)	10 (8–12)	11 (9–12)	7 (7–9)	9 (8–12)
**Female daily survival rate (%)**				
Mean ± Standard deviation	85.3 ± 19.0 A	91.9 ± 8.7 A	89.5 ± 12.1A	85.0 ± 17.5 A
**Male daily survival rate (%)**				
Mean ± Standard deviation	89.1 ± 15.1 A	90.2 ± 7.8 A	87.5 ± 18.5 A	89.7 ± 12.1 A

* Values marked by different letters within the same row, for the same species, are significantly different at a level of 0.05 (Tukey-Kramer HSD test).

## Discussion

The present study identified a significant effect of land use and land cover on vector survivorship. Mosquitoes placed under indoor environment exhibited significantly higher survivorship and longevity than banana plantation and forested environment. When mosquitoes were placed indoors in two sites differing in elevation, mosquitoes exhibited higher survivorship in sites with lower elevation. The effects of land use and land cover on mosquito survivorship likely resulted from differing microclimatic conditions among the habitats where adult mosquitoes were placed. Significantly higher mosquito survivorship was found in an indoor environment where mean daily temperature was 2°C higher than in the forested environment. This result on the impact of land use and land cover on mosquito survivorship was consistent with other studies on *An*. *arabiensis* and *An*. *gambiae* in African highlands [43, 44], and *An*. *darlingi* in the Peruvian Amazon [45].

The findings from this study have important implications for understanding malaria transmission and vector control in changing ecosystem. The developing world has been experiencing rapid land use and land cover changes. Deforestation is a major component of land use and land cover changes. Increased survivorship of adult mosquitoes in the indoor environment in deforested areas, as demonstrated in the present study, suggests that Indoor Residual Spraying (IRS) and Insecticide-Treated Nets (ITNs) should be used for vector control to prevent indoor malaria transmission. In addition, deforestation could alter the microclimatic conditions of aquatic habitats and subsequently enhanced survival and development of larval mosquitoes as demonstrated in *An*. *gambiae* and *An*. *arabiensis* in Africa [43, 46–48]. Because vector survivorship and vector density are important components of vectorial capacity, deforested agricultural areas could exhibit dramatically higher vectorial capacity than forested areas. Therefore, deforested agricultural area can increase the risk of malaria transmission.

There are several limitations in our study. First, although it is a conventional method, microcosm rearing of mosquitoes in cages for determination of vector survivorship was in a confined condition. In field conditions, mosquitoes could hide and rest in moisture and dry habitats with microclimate conditions that are different from our cage condition. Because it is not feasible to track the mosquitoes under field conditions, determination of vector survivorship under field conditions has been indirect based on biomarkers such as ovarian structural evaluation [49], fluorescent pigment pteridine concentration [50], cuticular hydrocarbon [51, 52], and gene expression [53]. These methods have significant limitation in estimation reliability such as the age of mosquitoes beyond certain period cannot be identified [54, 55], and sensitive to blood feeding and other physiological changes [56]. Our microcosm rearing of mosquitoes is the most direct measurement of mosquito survivorship. Second, we fed mouse blood and sucrose sugar in our experiments. The food source to adult mosquitoes may affect survivorship as *An*. *minimus* prefers biting human [57–59]. Because all mosquitoes were reared under the same food condition, the results on the impact of land use and land cover should be valid.

It is important to assess the impact of land use and land cover on vector-borne disease transmission when an economic development plan that significantly alters land use and land cover is being formulated. This study suggested that deforestation is the worst scenario, re-cultivation with banana plantation or other economically valuable trees such as rubber trees could boost incomes and reduce malaria transmission risk at the same time. Therefore, government policy should encourage local farmers to re-cultivate on deforested land. The estimated daily survival rate for *An*. *sinensis* and *An*. *minimus* under different land use and land covers provides a valuable parameter in modeling vector population dynamics and malaria transmission risk.

## Supporting Information

S1 Table Daily number of dead adult mosquitoes in different environments (Forest, Banana plantation, and Indoor) in China-Myanmar border region. (XLSX)

S2 Table Daily number of dead adult mosquitoes in different elevations (Daonong and Nabang) in China-Myanmar border region. (XLSX)
